# Compressed Sensing Reconstruction with Zero-Shot Self-Supervised Learning for High-Resolution MRI of Human Embryos

**DOI:** 10.3390/tomography11080088

**Published:** 2025-08-02

**Authors:** Kazuma Iwazaki, Naoto Fujita, Shigehito Yamada, Yasuhiko Terada

**Affiliations:** 1 Institute of Pure and Applied Sciences, University of Tsukuba, 1-1-1 Tennodai, Tsukuba, Ibaraki 305-8573, Japan; iwazakikazuma@gmail.com (K.I.); fujitan932@gmail.com (N.F.); 2 Congenital Anomaly Research Center, Graduate School of Medicine, Kyoto University, Yoshida-Konoe-cho, Sakyo-ku, Kyoto 606-8501, Japan

**Keywords:** high-resolution MR microscopy, human embryo, compressed sensing, deep learning reconstruction, spatial resolution

## Abstract

**Objectives**: This study investigates whether scan time in the high-resolution magnetic resonance imaging (MRI) of human embryos can be reduced without compromising spatial resolution by applying zero-shot self-supervised learning (ZS-SSL), a deep-learning-based reconstruction method. **Methods**: Simulations using a numerical phantom were conducted to evaluate spatial resolution across various acceleration factors (AF = 2, 4, 6, and 8) and signal-to-noise ratio (SNR) levels. Resolution was quantified using a blur-based estimation method based on the Sparrow criterion. ZS-SSL was compared to conventional compressed sensing (CS). Experimental imaging of a human embryo at Carnegie stage 21 was performed at a spatial resolution of (30 μm)^3^ using both retrospective and prospective undersampling at AF = 4 and 8. **Results**: ZS-SSL preserved spatial resolution more effectively than CS at low SNRs. At AF = 4, image quality was comparable to that of fully sampled data, while noticeable degradation occurred at AF = 8. Experimental validation confirmed these findings, with clear visualization of anatomical structures—such as the accessory nerve—at AF = 4; there was reduced structural clarity at AF = 8. **Conclusions**: ZS-SSL enables significant scan time reduction in high-resolution MRI of human embryos while maintaining spatial resolution at AF = 4, assuming an SNR above approximately 15. This trade-off between acceleration and image quality is particularly beneficial in studies with limited imaging time or specimen availability. The method facilitates the efficient acquisition of ultra-high-resolution data and supports future efforts to construct detailed developmental atlases.

## 1. Introduction

Magnetic resonance imaging (MRI) is a noninvasive technique that enables three-dimensional imaging with various contrast mechanisms. Among these, magnetic resonance microscopy (MRM)—a high-resolution form of MRI—is essential for accurately capturing the microstructural features of tissues and organs.

Recent efforts have focused on constructing three-dimensional volumetric atlases of human embryos. Traditionally, these efforts have relied on optical methods using serial histological sections. Human embryos are categorized into 23 Carnegie stages based on morphological differentiation [1]. During this period, major organ primordia form rapidly, rendering the embryo particularly susceptible to developmental abnormalities. Accordingly, understanding neurodevelopmental mechanisms during this phase is critical. Gasser et al. [2] digitized histological sections of human embryos obtained through optical microscopy and compiled a publicly accessible database containing approximately 12,991 section images and 3347 annotated images across all 23 Carnegie stages. Similarly, Bakker et al. [3] analyzed more than 15,000 histological sections to construct a 3D atlas covering Carnegie stages 7 through 23. However, histology-based approaches have inherent limitations. Accurate 3D reconstruction is often compromised by misalignment during sectioning, and the process demands extensive manual labor and expert resources.

To address these limitations, noninvasive MRI techniques have garnered increasing attention [4,5,6,7]. MRI offers the advantage of visualizing internal structures in detail while maintaining high spatial resolution. More importantly, it allows for continuous three-dimensional data acquisition without physically damaging the specimen. Previous studies have demonstrated the feasibility of constructing three-dimensional morphological models using MRI data [8,9]. Notably, in 2015, three-dimensional models of 101 human embryos spanning Carnegie stages 13–23 were successfully generated using MRI [8].

However, a high-resolution atlas based solely on MRI has yet to be constructed. Past studies aiming to build large-scale atlases using MRI data [8,9] employed MRM datasets with spatial resolutions no finer than (40 μm)^3^ [7], limiting their ability to model only relatively large anatomical structures such as brain vesicles and ventricles. As a result, the developmental processes of ultrastructural components—particularly within the central nervous system—remain largely unexplored.

One of the primary barriers to constructing a high-resolution MRI-based atlas is the significant increase in scan time. Spatial resolution in MRI is inherently three-dimensional; doubling the linear resolution reduces the voxel volume by a factor of eight. As voxel size decreases, the signal per voxel diminishes, resulting in a lower signal-to-noise ratio (SNR). To maintain adequate SNR, longer acquisition times are required, with the relationship governed by the following expression: SNR ∝ (voxel volume) × √(imaging time). This equation implies that doubling the linear spatial resolution necessitates a 64-fold increase in scan time to maintain the same SNR. To address the challenge of prolonged scan times in high-resolution imaging, a breakthrough was achieved in 2023: compressed sensing (CS) [10] was applied to accelerate imaging, enabling MRM at an isotropic resolution of (10 μm)^3^—the highest reported for human embryo imaging. CS exploits signal sparsity to reconstruct images from significantly fewer measurements than traditional sampling methods, thereby reducing the scan time. For instance, halving the number of sampled k-space points can reduce acquisition time by 50%. In a study by Makihara et al. [11], a twofold acceleration factor (AF = 2) successfully halved the imaging time; however, the acquisition still required approximately 12 days. In contrast, previous ex vivo high-resolution MRI studies have reported scan durations of 1–2 days per specimen [12,13,14], rendering the 12-day protocol exceptionally long. Such extended scan times are a considerable obstacle in human embryo imaging, as they hinder the acquisition of sufficient samples for morphometric analysis across various developmental stages. For studies of human embryos, capturing multiple developmental stages is essential. Consequently, prolonged scan durations are impractical from both logistical and resource perspectives. Therefore, reducing scan time beyond AF = 2 is critical to enabling the construction of a high-resolution embryonic atlas.

In recent years, research on accelerating clinical MRI acquisition has advanced considerably, driven in part by the need to reduce patient burden [15,16,17]. Undersampling (US) below the Nyquist criterion is commonly employed to shorten scan times, and CS-based reconstruction methods are typically used to reconstruct artifact-free images from US data. However, these methods face limitations, particularly when reconstructing high-quality images from datasets acquired using high acceleration factors (AFs) [18]. To address these challenges, deep learning (DL) techniques have been introduced to solve the US reconstruction problem. These approaches have shown the potential to increase AF while substantially preserving image quality, thus enabling significant reductions in scan time [19,20,21]. The most widely used framework is supervised learning (SL), in which fully sampled (FS) data satisfying the Nyquist criterion serve as the ground truth (GT). In this approach, paired FS and US datasets are used for training, allowing the DL model to learn a mapping from US inputs to their corresponding FS outputs. Alternatively, self-supervised learning (SSL) methods have been proposed, which generate GT-like targets directly from US data, thereby eliminating the need for separate FS datasets. However, both SL and SSL approaches generally require large volumes of training data [22]. Applying DL-based image reconstruction to human embryo imaging is a natural extension of these developments. However, recent studies have reported a decline in model performance when imaging conditions differ from those used during training [23]. This poses a significant challenge for embryo imaging, where high-resolution training data are scarce, making it difficult to develop robust DL models tailored for the task. To address this issue, Yaman et al. (2022) proposed a novel framework called zero-shot self-supervised learning (ZS-SSL) [24]. ZS-SSL enables image reconstruction using only the test data from a single scan, without the need for pretraining or external datasets. Notably, it has been shown to outperform traditional non-DL CS methods in producing high-quality reconstructions at higher AFs.

If ZS-SSL can facilitate the rapid imaging of human embryos, it may enable the construction of large-scale, high-resolution atlases. However, it is crucial that spatial resolution is preserved in embryo imaging, even at high acceleration levels. Despite the increasing interest in CS-based reconstruction methods—both traditional and DL-based—few studies have systematically investigated the relationship between AF and spatial resolution. Consequently, the extent to which acceleration can be applied without compromising resolution remains an open question.

In this study, we evaluated how ZS-SSL and other reconstruction methods affect spatial resolution as AF increases, using both simulation and experimental data. Based on the simulation results, we conducted validation experiments on human embryo imaging to assess the consistency between simulated performance and real-world results. Researchers typically assess image reconstruction quality using quantitative metrics such as the structural similarity index measure (SSIM) and peak signal-to-noise ratio (PSNR), in conjunction with visual inspection [25]. However, these conventional metrics do not always reflect actual spatial resolution, underscoring the need for a more direct evaluation method. To this end, we adopted a resolution estimation approach based on image blurring to assess the applicability of ZS-SSL in high-resolution imaging.

The contributions of this study are as follows:

- We applied deep-learning-based image reconstruction to high-resolution MRI of human embryos and demonstrated accelerated imaging at AF = 4 while preserving spatial resolution.
- We established a quantitative evaluation framework to assess the spatial resolution of the reconstructed MRM images.

## 2. Materials and Methods

### 2.1. Simulation Setup

In this study, simulations were conducted using a numerical phantom to evaluate whether spatial resolution degrades when image reconstruction is performed with ZS-SSL under US conditions. For comparison, zero-filled and conventional reconstruction methods were also tested. Because the resolution of CS-reconstructed images depends on the SNR, additional evaluations were performed under various SNR conditions.

**Figure 1:**
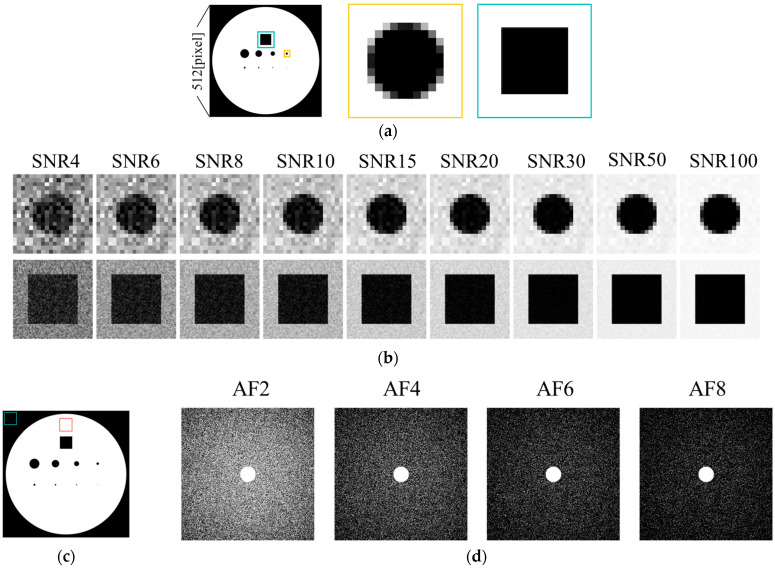
Numerical phantom used in the simulation. (**a**) Full view. (**b**) Simulated images with added Gaussian noise at various SNR levels. (**c**) ROI used for SNR calculation. (**d**) Undersampling patterns.

The numerical phantom used in this study is a two-dimensional image, as shown in Figure 1, consisting of circular and square structures of varying sizes. It was designed to simulate embedded structures in a solution-filled medium. The signal intensity of the solution was set to 1, while the background and embedded structures were set to 0. The phantom was generated as a square image with a resolution of *N* = 512 pixels. To simulate partial volume effects, an intermediate 4096 × 4096 pixel image was first generated, and the final 512-pixel image was obtained by averaging the signal intensities within each corresponding block of pixels, thereby preserving partial volume characteristics. The phantom contained nine structures: circular objects with diameters of 2, 4, 6, 8, 10, 20, 30, and 40 pixels, and one square object with a side length of 50 pixels (Figure 1a).

Images with different SNR levels were generated by adding random Gaussian noise to the k-space data obtained via Fourier transformation of the phantom image. This process yielded a set of images with nine SNR levels: 4, 6, 8, 10, 15, 20, 30, 50, and 100 (Figure 1b). The image SNR was calculated within a region of interest (ROI) as shown in Figure 1c. Specifically, the mean signal intensity of the target region *S_target_* and the standard deviation of the background *σ_b_* within the ROI were used to compute the SNR according to the following equation [26]:

(1) $$SNR=\sqrt{2-\frac{\pi }{2}}\;\frac{S_{target}}{\sigma_{b}}$$

### 2.2. Sampling Pattern

**Figure 2:**
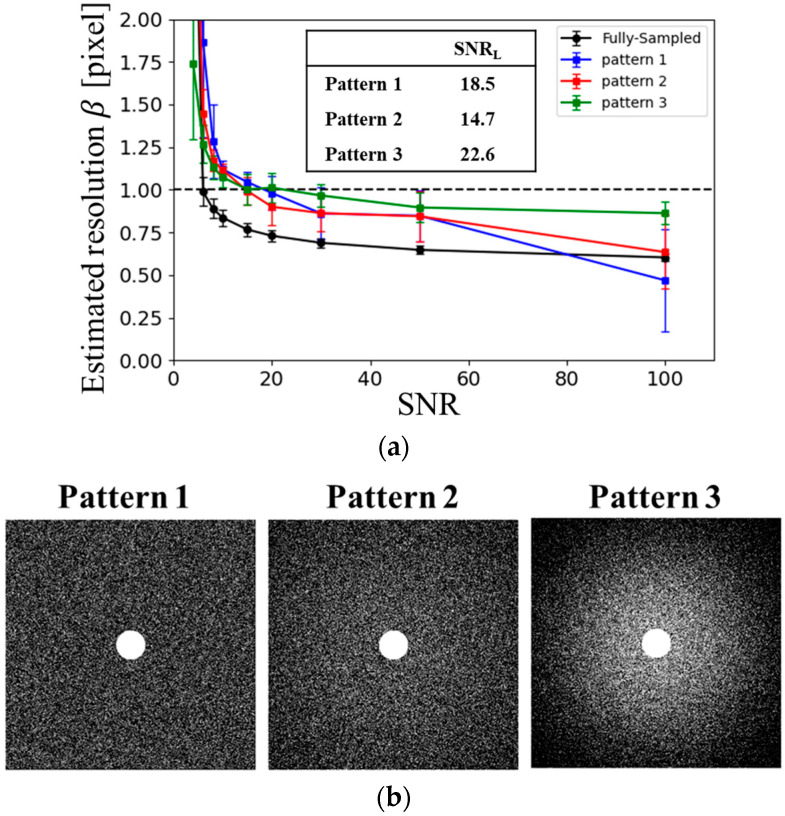
Sampling pattern dependence. (**a**) SNR dependence of the estimated resolution based on blur evaluation for different sampling patterns: Pattern 1 (blue), Pattern 2 (red), and Pattern 3 (green), under AF = 4. (**b**) Sampling patterns used in the analysis.

To evaluate the dependence of reconstruction performance on the US pattern, three distinct sampling schemes were prepared, as illustrated in Figure 2b. In all patterns, the central region of the k-space—within a circular area of radius 30 pixels—was fully sampled (i.e., Nyquist sampled). Random US was applied outside this region according to a predefined sampling probability density function *ρ*. Pattern 1 employed a uniform sampling density. In contrast, Patterns 2 and 3 used variable-density random sampling based on the probability function *ρ*, with different standard deviations *σ_s_*:

(2) $$\rho \propto \exp\left(-\frac{k_{x}^{2}+k_{y}^{2}}{2\sigma_{s}^{2}}\right)$$

In this study, the standard deviation *σ_s_* in the sampling probability function was defined as *σ_s_* = *k_max_* = 1/*N*, where *k_max_* denotes the maximum value of *k_x_*. Patterns 2 and 3 used *σ_s_* = 2 and *σ_s_* = 4, respectively.

### 2.3. Estimation of Resolution Using the Sparrow Criterion

In MRI, voxel size does not necessarily correspond to actual spatial resolution [27]. Therefore, the resolution of the reconstructed images was estimated using the Sparrow criterion [28]. For each reconstruction method, ten simulation trials were performed, varying both the sampling patterns and the random noise added to the k-space. The mean and variance of the estimated resolution were calculated. Resolution was defined using the method proposed by Chen et al. [29], which quantitatively evaluates image blurring as a proxy for spatial resolution. The noise-free phantom was treated as an ideal image *W(x, y)*. A blurred computational image, denoted *C(x, y, σ)*, was generated by convolving *W(x, y)* with a Gaussian function. The optimal blurring parameter *σ* was estimated by fitting *C(x, y, σ)* to the reconstructed image, and spatial resolution was then defined as *β* = 2*σ*. This definition follows the method proposed by Chen et al. [29], which estimates resolution by fitting reconstructed images to Gaussian-blurred versions of an ideal phantom. The Sparrow criterion was applied as a heuristic, using Gaussian blurring—not in its strict optical definition—to define resolution as 2*σ* of the fitted Gaussian point spread function (PSF), consistent with the approach in [29]. The convolution process is expressed as:

(3) $$C\left(x,y,\sigma \right)=\overset{w}{\underset{u=-w}{\sum }}\overset{w}{\underset{v=-w}{\sum }}W\left(x+u,y+v\right)\exp\left(-\frac{u^{2}+v^{2}}{2\sigma^{2}}\right)$$

where *w* denotes the kernel size and (*u,v*) are the coordinates within the kernel. Calculations were performed using the Gaussian filter function in the SciPy library [30] in Python. The kernel size matched the input image size, and edge handling was performed using the “nearest” mode, which interpolates using the nearest pixel value. The comparison between *C* and the reconstructed result followed Chen et al. [29], wherein *σ* was estimated by minimizing the sum of squared errors across all pixels.

### 2.4. Calculation of SNR_L_, the Lower SNR Limit for Resolution Preservation

The estimated resolution *β* was considered to match the nominal resolution when *β* was less than or equal to the voxel size, which was set to 1 pixel in this study. As discussed later, *β* increases as SNR decreases. Therefore, the SNR value at which *β* = 1 pixel was defined as the lower SNR limit (SNR_L_), below which spatial resolution could no longer be preserved. This value was determined by linearly interpolating the *β* versus SNR curve.

### 2.5. Reconstruction Methods

For CS reconstruction of US k-space data, we employed ZS-SSL [24], a DL-based image reconstruction method that does not require pretraining. Proposed by Yaman et al., ZS-SSL is a scan-specific DL approach tailored to individual acquisitions. In conventional DL-based CS reconstruction, a model is trained to map US data to FS data, and the trained model is then used to reconstruct new US inputs during inference. In contrast, the “zero-shot” aspect of ZS-SSL refers to its ability to perform reconstruction using only the k-space data from a single scan, without relying on external training datasets. In this method, the acquired k-space is divided into three mutually exclusive subsets. The first subset is used as input to train the DL model, the second is used to update model parameters, and the third serves as a validation set for early stopping to prevent overfitting.

**Figure 3:**
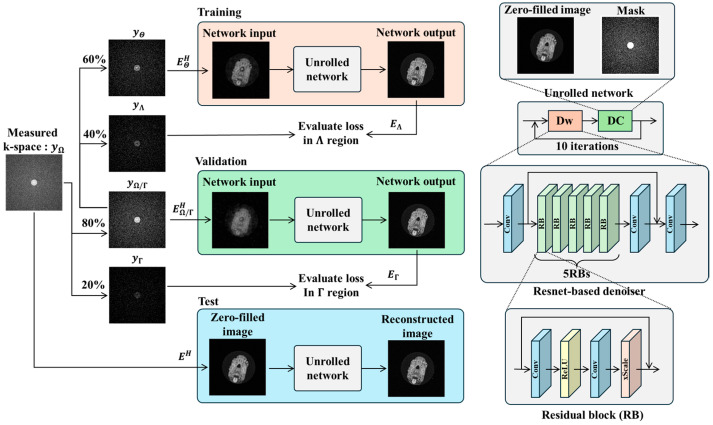
Workflow of zero-shot self-supervised learning (ZS-SSL) for MRI reconstruction. Measured k-space data from a single scan are split into three disjoint subsets: Θ (used for data consistency during training), Λ (used to compute training loss), and Γ (used to compute validation loss for early stopping). The unrolled network consists of 10 iterations of alternating data consistency (DC) and learnable denoising (Dw) units. The denoiser is a ResNet-based CNN composed of five residual blocks (RBs), each containing convolutional (Conv), ReLU, and scaling layers. During training, the network is updated using Θ and Λ. Validation loss is independently monitored using Γ, and training stops automatically when the loss increases over consecutive epochs. The final reconstruction is then performed using all available measurements and the trained network.

ZS-SSL was re-implemented based on Yaman et al. [24], using the PyTorch framework (PyTorch 2.1.0) [31]. The reconstruction network was built using a model-based deep learning (MoDL) approach [20], incorporating a ResNet architecture [32] (Figure 3). ResNet consists of five repeated residual blocks (RBs), each composed of a convolutional layer, a ReLU activation function, a second convolutional layer, and a scaling layer. The input and output of each RB are connected via a residual connection. The scaling layer multiplies the input by a factor α, which was set to 0.1 in this study. Each convolutional layer uses a 3 × 3 kernel and 64 filters. The number of reconstruction iterations was set to 10, and the learning rate was configured to 5 × 10^−4^. All other settings followed the original implementation by Yaman et al. [24]. In ZS-SSL, the measurement set Ω obtained from a single scan is partitioned as follows:

(4) Ω=Θ⊔Λ⊔Γ

where ⊔ represents disjoint union, and Ω, Λ, and Γ are pairwise disjoint subsets. The proportions of these subsets follow the data split illustrated in Figure 3. In ZS-SSL, the loss functions for training $L_{train}$ and validation $L_{val}$ are defined as follows:

(5) $$L_{train}=L\left(y_{\Lambda },E_{\Lambda }\left(f\left(y_{\Theta },E_{\Theta };\theta \right)\right)\right)$$

(6) $$L_{val}=L\left(y_{\Gamma },E_{\Gamma }\left(f\left(y_{\Omega \setminus \Gamma },E_{\Omega \setminus \Gamma };\theta \right)\right)\right)$$

Note that Ω∖Γ=Θ⊔Λ. The function $f\left(y,E;\theta \right)$ denotes the unrolled network, which takes the measured data *y* as input and uses an encoder operator E. $L_{train}$ is used to update the network parameters *θ*, whereas $L_{val}$ is used for early stopping. In ZS-SSL, both loss values are computed at each epoch. Training is terminated if $L_{train}$ > $L_{val}$ for 20 consecutive epochs. After early stopping, the entire measured k-space dataset is passed to the trained network to generate the final reconstructed image. The loss function $L\left(u,v\right)$ used in this study combines L1 and L2 norms and is defined as:

(7) $$L\left(u,v\right)=\frac{{\left\|u-v\right\|}_{1}}{{\left\|u\right\|}_{1}}+\frac{{\left\|u-v\right\|}_{2}}{{\left\|u\right\|}_{2}}$$

where u and v are arbitrary vectors.

To evaluate the performance of ZS-SSL, we compared it with two representative reconstruction methods: ConvDecoder [33] and conventional CS. ConvDecoder is a scan-specific CNN-based reconstruction framework proposed by Darestani et al. In this study, we used the publicly available implementation provided by the authors on GitHub (https://github.com/MLI-lab/ConvDecoder, commit db3a13c, accessed on 31 May 2025) to reconstruct images from undersampled data. For further comparison, we also evaluated image resolution using conventional CS reconstruction, implemented with L1-wavelet regularization, similar to the approach proposed by Lustig et al. [10]. The regularization weight was set to 2 × 10^−3^, and the implementation was conducted using the SigPy library in Python [34].

All computations were performed using Python version 3 (Python Software Foundation, Wilmington, DE, USA). The software environment was managed within a virtual container using Docker Engine. The hardware setup included a Windows 11 Pro system equipped with two GPUs: NVIDIA GeForce RTX 3080 (12 GB RAM) and an NVIDIA GeForce RTX 3090 (24 GB RAM).

### 2.6. Embryo Specimen

**Figure 4:**
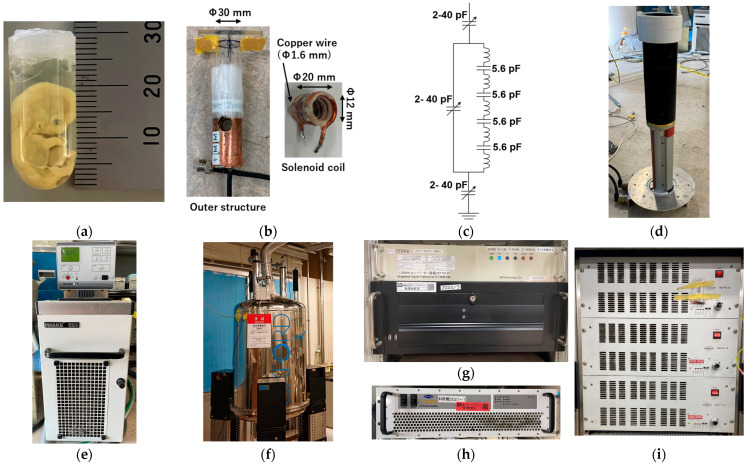
MR microscopy system. (**a**) Carnegie stage 21 human embryo specimen. (**b**,**c**) RF coil and resonance circuit. (**d**) Gradient coil. (**e**) Recirculating chiller. (**f**) Vertical superconducting magnet. (**g**) Transceiver and measurement PC. (**h**) RF amplifier. (**i**) Gradient amplifier.

In this study, a human embryo at Carnegie stage 21 (Figure 4a) was imaged using MRM. Embryonic specimens were provided by the Congenital Anomaly Research Center, Graduate School of Medicine, Kyoto University, Japan. These specimens are part of the Kyoto Collection [35,36], systematically assembled in Japan between 1961 and 1974. Embryos were chemically fixed in Bouin’s solution and preserved in 10% formalin. For imaging, the specimen was placed in a test tube with an outer diameter of 12.0 mm and immobilized using 1% agarose gel. The experimental use of human embryos was approved by the Ethics Committee of Kyoto University.

### 2.7. MR Microscopy System

The radiofrequency (RF) coil was custom designed to match the size of the embryo specimens (Figure 4b). It was used for both signal transmission and reception. The shielding box was fabricated by wrapping copper foil around an acrylic tube with an outer diameter of 20 mm. The coil consisted of four turns of copper wire (1.6 mm diameter) with an inner diameter of 10 mm. It was segmented into five sections using 5.6 pF chip capacitors (Figure 4b,c). A tunable capacitor (2–40 pF, AP40HV, Knowles Voltronics, NJ, USA) was used to tune and match the tank circuits.

The gradient coil (micro2.5, Bruker, Billerica, MA, USA) (Figure 4d) had an inner diameter of 41 mm, an outer diameter of 73 mm, and a maximum gradient strength of 25 mT/m (2.5 G/cm). To prevent overheating, temperature-controlled water (20 °C) was circulated within the gradient system using a recirculating chiller (Haake F6-C25, HAAKE Technil GmbH, Vreden, Germany) (Figure 4e).

Experiments were conducted using an MRI system equipped with a 9.4 T vertical wide-bore (89 mm diameter) superconducting magnet (Oxford Instruments, Abingdon, UK) (Figure 4f). The digital MRI console included a digital transceiver (DTRX6, MR Technology, Tsukuba, Japan) (Figure 4g), a preamplifier with a noise figure of 0.5 dB and a gain of 30 dB (DST Inc., Asaka, Japan) (Figure 4h), and a gradient driver (BAFPA40, Bruker, Billerica, MA, USA) (Figure 4i).

### 2.8. Imaging Sequence

Imaging was performed using a steady-state free precession free induction decay (SSFP-FID)-based three-dimensional gradient echo sequence with the following parameters: TR/TE = 100/5 ms, flip angle = 70°, field of view (FOV) = 2.1 × 1.62 × 1.62 cm^3^, and matrix size = 700 × 560 × 560, yielding an isotropic spatial resolution of (30 μm)^3^. Under FS conditions with a number of excitations (NEX) = 1, the total scan time was 8.7 h. For actual embryo imaging, NEX was increased to 4 to ensure sufficient SNR. Accelerated imaging was performed at AFs of 4 and 8, and FS imaging was conducted for comparison.

### 2.9. Retrospective and Prospective Study

In MRI, eddy currents generated during the rapid switching of gradient magnetic fields can distort the gradient waveform, potentially resulting in deviations from the intended US pattern. This effect poses challenges in prospective studies, where the actual sampling pattern may differ from the design. In contrast, retrospective studies apply US patterns to FS data during post-processing, allowing reconstruction performance to be evaluated independently of hardware-induced distortions. In this study, we compared retrospective and prospective approaches for human embryo imaging. The results were evaluated based on line profile analysis and resolution estimation using a blur-based method.

## 3. Results

### 3.1. Simulation of Resolution Estimation Using the Sparrow Criterion

Figure 2 presents the estimated spatial resolution for different sampling patterns at an AF of 4. Among the patterns tested, Pattern 2 yielded the lowest lower limit of signal-to-noise ratio (SNR_L_) required to preserve resolution. Based on these findings, Pattern 2 was selected as the optimal sampling pattern for this study.

**Figure 5:**
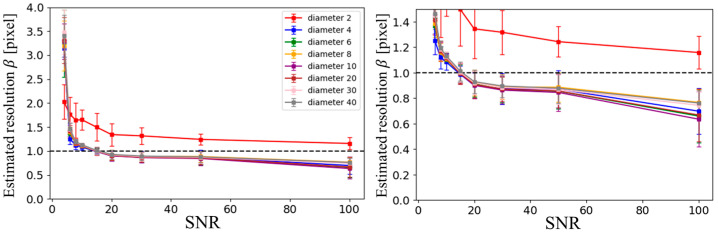
SNR dependence of estimated resolution based on blur evaluation in ZS-SSL–reconstructed images at AF = 4, across different circular structure sizes. The phantom consists of circles with diameters of 2, 4, 6, 8, 10, 20, 30, and 40 pixels. The left panel displays the full range of resolution estimates, while the right panel provides a magnified view for better comparison at lower resolution values.

Figure 5 shows the estimated resolution, based on the Sparrow criterion, for ZS-SSL reconstruction at AF = 4. As illustrated, except for the smallest structure (2-pixel-diameter circle), the estimated resolution showed little variation across different circular structure sizes. Consequently, in subsequent analyses, the results for the 20-pixel-diameter circle were used as representative data.

**Figure 6:**
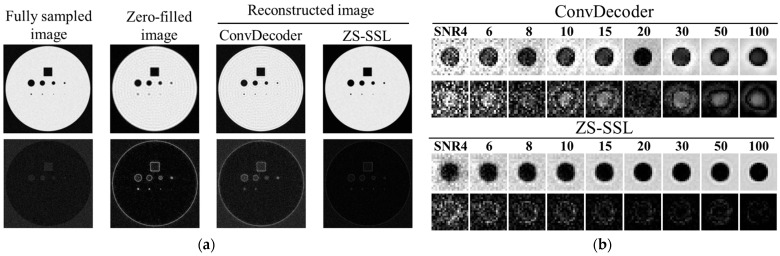
Comparison of reconstruction results between ZS-SSL and ConvDecoder. Difference images from the noise-free reference (Figure 1a) are shown below each reconstructed image. The intensity of the difference images is scaled by a factor of 2. (**a**) Reconstructed images and corresponding difference maps under representative conditions (AF = 4, SNR = 15). Fully sampled and zero-filled images are also shown for reference. (**b**) Reconstruction results at different SNR levels for each method.

Figure 6a compares the reconstruction results obtained using ConvDecoder and ZS-SSL. In the ConvDecoder output, artifacts from the zero-filled image remained visible, and the difference image indicated incomplete reconstruction. In contrast, ZS-SSL effectively suppressed these artifacts, with the difference image showing a reconstruction quality comparable to the fully sampled image. Figure 6b focuses on reconstructing a small structure with a diameter of 10 pixels. Compared to ConvDecoder, ZS-SSL resulted in lower reconstruction errors, indicating superior structural fidelity.

**Figure 7:**
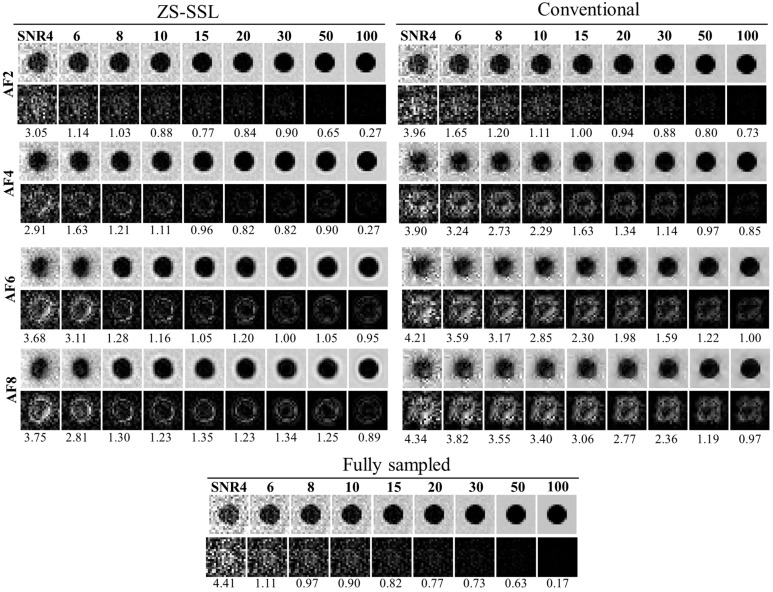
Blur-based resolution estimation for reconstructed images using ZS-SSL and conventional CS. The FS image is also shown for comparison. The structure has a diameter of 10 pixels. Note the increase in image blurring as AF increases, especially at AF = 6 and 8, where boundary clarity is reduced even at high SNR. Difference images from the noise-free reference (Figure 1a) are shown below each reconstruction, with intensities scaled by a factor of 2.

Simulated images of the 10-pixel-diameter circle, including the FS image for comparison, are shown in Figure 7. In both ZS-SSL and conventional CS methods, image blurring along the circular boundary increased as AF and SNR decreased. At AF = 6 and 8, boundary blurring was apparent across all SNR levels. To visualize reconstruction errors, difference images relative to the noise-free binary phantom (Figure 1a), scaled in intensity by a factor of 2, are displayed below each reconstruction. These difference images demonstrate that ZS-SSL preserves more structural detail than conventional CS, particularly at AF = 4. However, structural degradation becomes evident for ZS-SSL at AF = 6 and 8, while conventional CS shows substantial reconstruction errors even at AF = 4, worsening further with higher AF.

**Figure 8:**
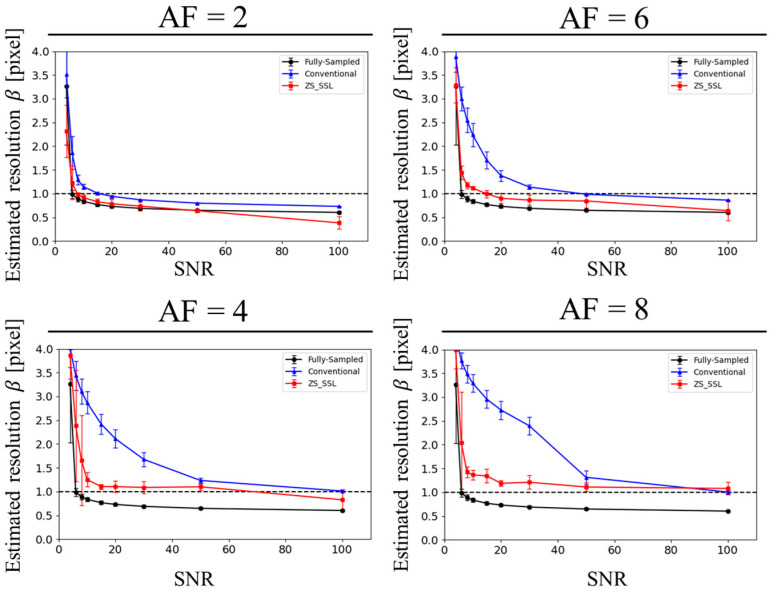
SNR dependence of the estimated resolution (*β*) based on blur evaluation for circular structures with a radius of 10 pixels. Reconstruction methods include FS (black), conventional CS (blue), and ZS-SSL (red). Results from 10 trials are shown for each method, with mean resolution (circles) and variance (error bars). AF conditions are labeled at the top of each plot. Note the marked resolution loss at higher AFs (AF = 6 and 8), especially under low SNR conditions.

Figure 8 presents the estimated resolution values (*β*) across different SNR levels. For the same reconstruction method and AF, *β* increased as SNR decreased, indicating a decline in resolution. Across AFs, *β* generally rose with higher AFs. At a fixed SNR, the *β* value was highest for conventional CS, followed by ZS-SSL, and lowest for FS, reflecting the expected hierarchy in reconstruction performance.

The lower limit of SNR required to maintain spatial resolution (SNR_L_) is summarized in Table 1. Consistent with the visual results, SNR_L_ increased with higher AF, indicating that greater SNR is necessary to maintain target resolution under stronger undersampling. At the same AF, ZS-SS_L_ consistently achieved lower SNR_L_ values than conventional CS, suggesting superior reconstruction performance under low-SNR conditions. Clear SNR_L_ values were identifiable for AF ≤ 4; however, for AF ≥ 6, they were undefined in most cases. Notably, at AF = 6, ZS-SSL required an SNR_L_ of 68.4, which is difficult to achieve in high-resolution MRI. These findings suggest that, at AF ≥ 6, maintaining sufficient SNR for acceptable resolution becomes impractical, rendering such AFs unsuitable for routine imaging applications.

### 3.2. Experimental Protocol for Accelerated Imaging of Human Embryos

**Figure 9:**
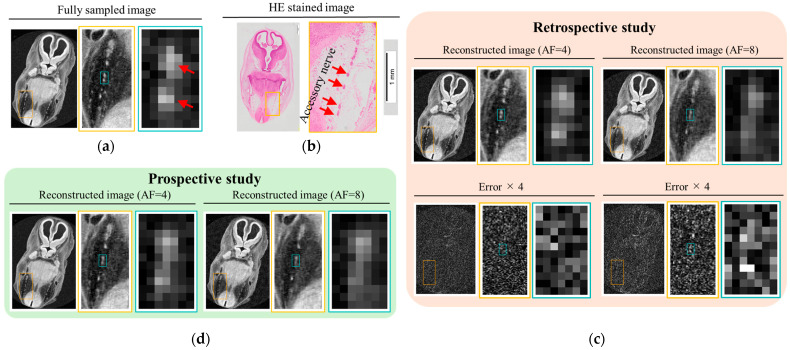
Cross-sectional images perpendicular to the course of the accessory nerve, acquired using fully sampled imaging at a spatial resolution of (30 μm)^3^. (**a**) FS image of a human embryo at Carnegie stage 21. (**b**) HE-stained image of a different embryo at the same stage, shown for comparison. (**c**,**d**) Reconstructed images from retrospective (**c**) and prospective (**d**) studies at AF = 4 and AF = 8. In (**c**), difference images relative to the FS reference are shown below each reconstruction, with intensities scaled by a factor of 4.

Figure 9a shows a cross-sectional image, perpendicular to the course of the accessory nerve, obtained from an FS MRI scan of a human embryo at a spatial resolution of (30 μm)^3^. Images are displayed from left to right with increasing magnification. The SNR of the FS image is approximately 19. In the corresponding histological image (Figure 9b), the red arrow indicates the location of the accessory nerve. Reconstructed images from the retrospective (Figure 9c) and prospective (Figure 9d) studies are displayed in the same manner for direct comparison with the FS image.

At this developmental stage, the accessory nerve has an approximate diameter of 100 μm. In the FS image, the nerve is clearly visualized, consistent with the histological reference. In the retrospective study, the accessory nerve remained clearly distinguishable at AF = 4, with sufficient contrast to differentiate it from surrounding structures. The corresponding difference image showed minimal residual structural information, indicating an accurate reconstruction. However, at AF = 8, although a nerve-like structure could still be identified, it was difficult to differentiate it from adjacent tissues. The corresponding difference images revealed visible structural components, suggesting the presence of anatomical reconstruction errors. A similar trend was observed in the prospective study: while the accessory nerve was still distinguishable at AF = 4, its visibility diminished at AF = 8.

**Figure 10:**
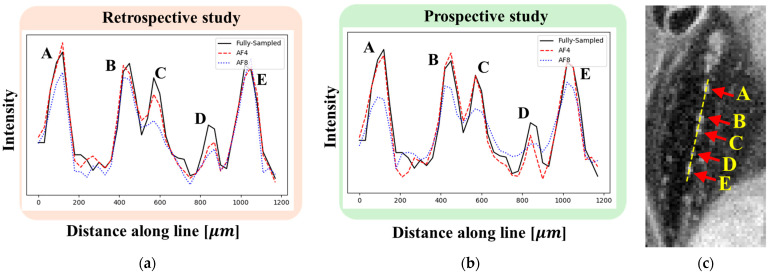
Line profiles perpendicular to the course of five accessory nerves. (**a**,**b**) Line profiles from retrospective (**a**) and prospective (**b**) studies at AF = 4 (red) and AF = 8 (blue). For comparison, the FS line profile is also shown (black). (**c**) FS image indicating the locations of the five accessory nerves.

Line profiles were obtained perpendicular to the course of five accessory nerves, labeled A–E (Figure 10). In the FS image, distinct signal peaks corresponding to nerves A–E were clearly observed. At AF = 4, reconstructions from both retrospective and prospective studies yielded line profiles similar to the FS image. In contrast, at AF = 8, the signal intensities for nerves C and D decreased in the retrospective reconstruction, while, in the prospective reconstruction, reduced signal intensities were observed across all five nerves (A–E). Notably, the signal drop at AF = 8 made it difficult to distinguish between the closely spaced nerves B and C, indicating incomplete structural reconstruction.

**Figure 11:**
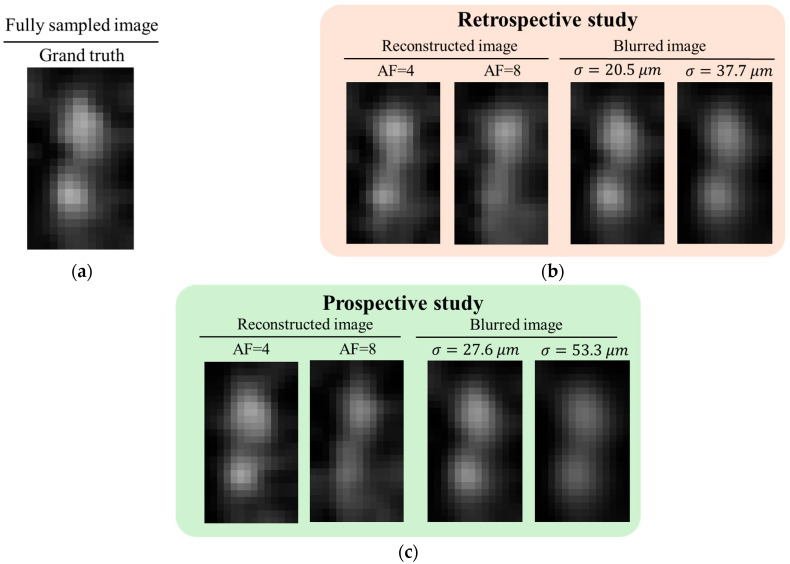
Quantitative evaluation of structural degradation in ZS-SSL–reconstructed images at AF = 4 and AF = 8, using the FS image as GT. (**a**) Upsampled FS image of the accessory nerve (bilinear interpolation, ×2 in each dimension), used as the reference for blur fitting. (**b**,**c**) Retrospective (**b**) and prospective (**c**) ZS-SSL reconstructed image (left) with corresponding Gaussian-blurred version of GT (left). *σ* indicates the estimated blur level. Resolution was defined as 2*σ*’(*σ*’ = 0.5*σ*), following the convention used in [29].

Figure 11 presents a quantitative evaluation of structural degradation in ZS-SSL–reconstructed images at AF = 4 and AF = 8, using the FS image as the GT. Assuming a roughly circular structure for the accessory nerve, resolution was estimated using the blur-based method described in Figure 7. To reduce estimation errors caused by the limited number of pixels representing the target structure, bilinear interpolation was applied to double the image matrix size in each linear dimension. In Figure 11, both the upsampled reconstructed images and the corresponding blurred images—obtained by applying a Gaussian filter with the estimated blur parameter *σ* to the FS image—are shown. In the retrospective study, the estimated *σ* was 20.5 μm at AF = 4 and 37.7 μm at AF = 8. In the prospective study, *σ* was 27.6 μm at AF = 4 and 53.3 μm at AF = 8. The actual blur size was taken as *σ*’ = 0.5*σ*, and the resolution was defined as 2*σ*’, following the Sparrow criterion. At AF = 4, both retrospective and prospective reconstructions yielded resolutions below the nominal imaging resolution of 30 μm, indicating preservation of spatial resolution. In contrast, at AF = 8, both cases exhibited resolutions exceeding 30 μm, reflecting a decline in spatial resolution.

## 4. Discussion

### 4.1. Research Summary

This study addressed the challenge of prolonged acquisition times in high-resolution MRI of human embryos by introducing ZS-SSL and validating its effectiveness through simulations and experimental imaging. The results demonstrate that ZS-SSL enables accelerated imaging at an AF = 4 while preserving spatial resolution, achieving a practical balance between scan duration and image quality. This is particularly important given the rarity of human embryo specimens, for which dataset-dependent models are often impractical. While Makihara et al. [11] achieved ultra-high-resolution MRI at (10 μm)^3^ using a two-fold acceleration (AF = 2), their protocol required extensive scan times. In contrast, the present study demonstrated stable reconstruction at AF = 4 while preserving fine anatomical details at a resolution of (20 μm)^3^. This highlights the advantage of ZS-SSL in enabling higher acceleration without the need for extensive pretraining or large training datasets.

The phantom experiments confirmed that ZS-SSL outperforms the state-of-the-art ConvDecoder method for scan-specific reconstruction. One limitation of ConvDecoder is its rigid network architecture, which supports only fixed image matrix sizes. In this study, matrix sizes differed between the phantom and embryo datasets. Consequently, reconstructing embryo data using ConvDecoder would have required retraining the network architecture, posing a significant constraint. Furthermore, ConvDecoder is highly sensitive to network configuration and demands advanced hyperparameter tuning. In contrast, ZS-SSL is free from these constraints, surpasses ConvDecoder in phantom reconstruction, and offers greater overall robustness and flexibility.

To evaluate spatial resolution degradation—a dimension often inadequately captured by conventional image quality metrics—we introduced a novel blur-based resolution estimation method based on the Sparrow criterion. This framework effectively visualizes and quantifies resolution loss and has the potential to be applied across various high-resolution image reconstruction tasks as a general and practical evaluation tool.

Furthermore, our findings indicate that ultra-high-resolution imaging at (20 μm)^3^ can be achieved within a realistic scan time of approximately four days under AF = 4 conditions. This capability offers a valuable foundation for comprehensive morphological analyses across multiple developmental stages and represents a significant technical advancement in the development of high-precision MRI-based developmental atlases.

### 4.2. Potential Enhancements of ZS-SSL in High-Resolution MRI

ZS-SSL, a deep-learning-based image reconstruction method, offers a compelling solution to the trade-off between spatial resolution and acquisition time by enabling significant scan time reductions. Its ability to learn directly from a single scan without relying on external datasets makes it particularly suitable for MRM applications, where available data are often limited. Although ZS-SSL achieved stable reconstructions up to AF = 4, image degradation became apparent at AF = 8. Recent studies have shown that incorporating multiple contrast images as multichannel inputs—such as T1-, T2-, and T2*-weighted images—can enhance performance in deep-learning-based reconstruction by increasing contrast sensitivity and reducing undersampling artifacts [36]. These benefits have primarily been demonstrated in in vivo imaging, where structural redundancy across contrasts plays a key role [36]. However, such redundancy may also be present in ex vivo imaging. Consequently, similar multichannel strategies could potentially enhance effective resolution and allow for higher AFs in ZS-SSL frameworks used in ex vivo applications, including studies involving human embryos.

Integrating these strategies with the current ZS-SSL framework could further improve both resolution and reconstruction speed, facilitating broader applications in research areas that demand high-resolution and efficient morphological imaging. These include the development of embryonic atlases and the detailed structural analysis of developmental processes.

### 4.3. Framework for Assessing Reconstruction Resolution in MRM

In MRI, it is well known that the image matrix size does not necessarily reflect the actual spatial resolution [27], as image quality is significantly influenced by the reconstruction algorithm and noise levels. Huang et al. [37] reported that deep-learning-based reconstructions are particularly susceptible to excessive smoothing under high-noise conditions, potentially obscuring or degrading structural information. This effect is especially critical in high-resolution imaging, where fine anatomical details may be lost, compromising the accuracy of morphological analyses—an especially concerning issue in studies of human embryos. Therefore, quantitatively assessing the relationship between spatial resolution and noise levels is crucial to determining optimal imaging conditions. To this end, we developed a practical framework to evaluate the interdependence of US, SNR, and spatial resolution using a blur-based estimation approach grounded in the Sparrow criterion. Unlike traditional metrics such as the structural similarity index (SSIM) or PSNR, this method directly quantifies resolution degradation. As such, it offers a generalizable and reliable evaluation tool for high-resolution MRI applications beyond the present study.

### 4.4. Future Perspectives on Constructing Developmental Atlases Based on High-Resolution MRI

This study demonstrated that accelerated imaging using ZS-SSL at AF = 4 achieves twice the imaging efficiency of previous methods. As a result, it is now feasible to image multiple human embryo specimens within a limited scan time, laying the groundwork for capturing developmental changes across various stages with both high precision and coverage. Notably, imaging and analysis of multiple specimens across seven Carnegie stages (from stage 17 to 23, excluding stage 16) have become realistic and meaningful objectives. Such efforts would enable the construction of a high-resolution developmental atlas that reflects inter-individual variability, advancing our understanding of human embryogenesis.

Notably, an SNR_L_ of 14.7 at AF = 4 was confirmed to be practically achievable under the experimental conditions. For the Carnegie stage 21 embryo, the fully sampled image acquired at a spatial resolution of 30 μm with NEX = 4 achieved an SNR of approximately 19. Under the same conditions, the AF = 4 reconstructed image maintained the clear visibility of anatomical structures (see Figure 9 and Figure 11). These findings suggest that, with the current imaging setup, high-resolution imaging at AF = 4 is feasible for embryos up to Carnegie stage 21. For later stages, such as Carnegie stages 22 and 23, the specimens could not be accommodated within the existing 12 mm diameter tube, necessitating the use of a larger tube (approximately 15 mm in diameter) and a corresponding RF coil. While changes to the imaging sequence are not required, the increased coil diameter may reduce sensitivity, and achieving SNR_L_ = 14.7 may require a longer scan time through increased NEX or other adjustments.

Looking forward, a promising extension of ZS-SSL is the incorporation of multicontrast images as multichannel inputs into the reconstruction network. This approach is expected to not only enable acceleration beyond AF = 4 but also to improve imaging throughput. Moreover, the complementary information provided by different contrasts may further enhance reconstruction accuracy. For example, Kunieda et al. [38] demonstrated that anatomical structures difficult to visualize with conventional T1-weighted imaging—the predominant modality in human embryo MRI—can be more clearly depicted using alternative contrasts such as T2*-weighted imaging. These results suggest that multicontrast imaging could enhance tissue-specific visibility and facilitate the construction of more information-rich three-dimensional atlases. Thus, the advancement of multicontrast strategies represents both a key challenge and an essential direction for future developmental atlas research.

To support this effort, high-resolution MRI scans of multiple human embryos at each Carnegie stage are needed to statistically evaluate inter-individual variability. For instance, following the approach proposed by Kishimoto et al. [39], principal component analysis (PCA) could be applied independently to each Carnegie stage to extract the major axes of morphological variation. This would enable the construction of spatiotemporal statistical models based on high-resolution MRI data. Such models can provide detailed insights into fine-scale growth patterns during embryonic development and may also support the early detection and quantitative assessment of structural abnormalities. Ultimately, this study contributes to the development of a practical and comprehensive 3D atlas that integrates morphological information across developmental stages, forming a robust foundation for advancing knowledge in human embryogenesis.

### 4.5. Potential Applications in Magnetic Resonance Spectroscopy

The integrated approach presented in this study—combining a high-performance self-supervised reconstruction method with quantitative phantom-based resolution assessment—may have applications beyond fast MRI. It can potentially be extended to fields such as NMR spectroscopy [40,41,42] and magnetic resonance spectroscopic imaging (MRSI) [43,44,45]. For example, in fast NMR spectroscopy, non-uniform sampling (NUS) is commonly used to reduce scan times. However, a key challenge remains: objectively evaluating whether the reconstruction algorithm compromises true spectral resolution, such as by merging closely spaced peaks, in favor of improved apparent SNR [46]. The methodology proposed in this study could serve as a systematic solution. Specifically, by using a standard sample with known, closely spaced peaks, reconstruction performance can be objectively evaluated by comparing metrics such as peak separation and linewidth in spectra reconstructed from NUS data versus those obtained through conventional uniform sampling.

In this context, a scan-specific self-supervised learning method such as ZS-SSL—which does not require extensive pretrained datasets—may offer greater versatility and reliability than existing deep learning models, especially for the diverse molecules and experimental conditions encountered in NMR. Moreover, the methodology is applicable to the more complex domain of MRSI, where achieving high resolution in both spatial and spectral dimensions is essential.

### 4.6. Limitations

This study had several limitations. First, because the objective was to construct a high-resolution atlas of human embryos—a rare and valuable specimen type—ZS-SSL, which does not require pretraining, was particularly well suited to the nature of the dataset. However, in model organisms such as mice, where large high-resolution datasets are more readily available, supervised or conventional self-supervised learning approaches [47] could potentially yield better reconstruction performance. Second, our simulations did not account for the potential influence of matrix size. Specifically, the impact of varying FOV or resolution—both of which alter the matrix size—on ZS-SSL reconstruction performance, especially in terms of spatial resolution, remains to be explored in future work. Third, the optimization of sampling patterns was limited in scope. While we evaluated performance using three types of sampling patterns, further exploration may lead to improvements, such as supporting higher AFs or reducing the required SNR (SNR_L_).

## 5. Conclusions

In this study, we investigated the effects of aggressive US on spatial resolution in accelerated MRI reconstruction using ZS-SSL. The results demonstrate that ZS-SSL can maintain sufficient resolution at AF = 4; however, image degradation becomes evident at AF = 8. The methodology presented offers a generalizable approach for evaluating resolution in accelerated MRI and holds potential for broader application, including the high-resolution microscopy of rare or delicate specimens. Future integration with multicontrast imaging and learned sampling strategies could unlock further improvements in terms of both reconstruction speed and fidelity.

## Figures and Tables

**Table 1:** Lower SNR limit (SNR_L_) required to preserve spatial resolution.

	AF = 1	AF = 2	AF = 4	AF = 6	AF = 8
FS	6.0	-	-	-	-
CS	-	15.6	48.1	NA	NA
ZS-SSL	-	7.9	14.7	64.8	NA

## Data Availability

The code used in this study for zero-shot self-supervised MRI reconstruction is publicly available at: https://github.com/mrlab-tsukuba/zs_ssl_recon2025 (commit 4a94622, accessed on 31 May 2025). The human embryo MRI data are not publicly available due to ethical restrictions.

## Abbreviations

The following abbreviations are used in this manuscript:

MRI	magnetic resonance imaging
MRM	magnetic resonance microscopy
SNR	signal-to-noise ratio
CS	compressed sensing
US	undersampling
FS	fully sampled
AF	acceleration factor
DL	deep learning
SL	supervised learning
SSL	self-supervised learning
GT	ground truth
ZS-SSL	zero-shot self-supervised learning
SSIM	structural similarity index measure
PSNR	peak signal-to-noise ratio
SNR_L_	signal-to-noise ratio limit
FOV	field of view
